# Structural Equation Modelling of Retinopathy of Prematurity Treatment Integrating Both Physical and Clinical Effects

**DOI:** 10.3390/jcm14020297

**Published:** 2025-01-07

**Authors:** José Luis García-Serrano, Olena Protsyk, Teresa Domech-Serrano, José Uberos Fernández

**Affiliations:** 1 Department of Surgery and Related Specialties, University of Granada, 18012 Granada, Spain; 2 Ophthalmology Service, San Cecilio Clinical Hospital, 18016 Granada, Spain; teresa.domech.sspa@juntadeandalucia.es; 3 Department of Ophthalmology, Jaen University Hospital, 23007 Jaén, Spain; olenaprotsyk@correo.ugr.es; 4 Neonatal Intensive Care Unit, Medicine Faculty, San Cecilio Clinical Hospital, 18016 Granada, Spain; juberos@ugr.es

**Keywords:** retinopathy of prematurity, therapy, structural equation models, risk factors, aetiology, physiopathology, peripheral avascular retina

## Abstract

**Background:** We sought to develop a structural equation model (SEM) identifying physical and clinical risk factors associated with treatment for retinopathy of prematurity (ROP). **Methods:** This retrospective, observational, case–control study included 314 infants screened for ROP between April 2004 and July 2024. A bivariate binary logistic regression model, decision tree, and structural equation model (SEM) were employed to develop a more general model for ROP requiring treatment. **Results:** In the SEM, the factors significantly associated with ROP treatment included the retinal avascular area according to disk diameter (DD) (*p* < 0.001), weekly vascularisation rate (DD/w) (*p* < 0.001), and duration of intubation (days) (*p* < 0.001). In addition, the following significant associations were identified in both the bivariate analysis and the SEM: lower gestational age (*p* < 0.001) and birth weight (*p* <0.001) were associated with greater retinal avascular area; low postnatal weight gain (*p* < 0.027) was associated with a slow rate of retinal vascularisation; sepsis (*p* < 0.001), ductus arteriosus (*p* < 0.001), and the need for transfusion (*p* < 0.001) were associated with longer intubation mechanical ventilation (IMV). **Conclusions:** Lower gestational age, lower birth weight, sepsis, ductus arteriosus, transfusion, and lower weight gain increase the risk of requiring ROP treatment. In the SEM, this association is represented through three intermediate physical endogenous variables, namely, the greater temporal avascular area of the retina, the lower postnatal vascularisation rate, and the greater duration of IMV.

## 1. Introduction

Occurring on the immature and incompletely vascularised retinas of premature infants, retinopathy of prematurity (ROP) is a developmental neurovascular disorder characterised by abnormal blood vessels growing in the retina. Retinal vascularisation in premature infants usually presents three patterns: (a) normal retinal angiogenesis is completed between 20 and 40 weeks; (b) disordered and delayed vascularisation can be divided into five stages of development of classical ROP, with stages 4 and 5 of ROP potentially leading to retinal detachment and blindness; and (c) the appearance of aggressive posterior retinopathy of prematurity (AP-ROP) and the less frequent ROP variant, which causes early pathologic neovascularisation located in zone I or posterior zone II, and this disease may rapidly progress to stage 5 ROP.

ROP causes blindness in 30,000 children worldwide annually. Continuous and regular examinations of the retina by an ophthalmologist, called screenings, are necessary for all prematurely born infants. These screenings allow for the early diagnosis and treatment of ROP, with a good response to treatment in 90–95% of premature infants. Knowing the modifiable risk factors for ROP allows us to act at two levels: general-paediatric and local-ocular.

Several risk factors contribute to the need for ROP treatment [1,2]. Among these, low gestational age and birth weight are primary inclusion criteria for ROP screening and treatment risk confirmation [3]. Oxygen exposure in neonatal intensive care units (NICUs) is another significant risk factor, potentially linked to severe ROP development [4]. Numerous other clinical prognostic factors have also been included in predictive models, including low postnatal weight gain, sepsis [5], persistent ductus arteriosus, a low Apgar score at 1 and 5 minutes after birth, transfusion, anaemia, thrombocytopenia, and bronchopulmonary dysplasia.

In our predictive logistic regression model for ROP treatment, only two physical variables, peripheral avascular area and weekly vascularisation rate, and one clinical variable, the duration of intubation mechanical ventilation (IMV), were found to be significant. In the initial screening, if the peripheral avascular retina (PAR) is very extensive, with 6–7 papillary disc diameters (DD), ROP treatment is usually required. If the PAR ranges from 3–6 DD, a higher vascularisation velocity (>0.5–0.54 DD/week) may facilitate temporary peripheral vascularisation and prevent progression to type I ROP [6,7]. Extended IMV increases the risk of severe ROP. However, our predictive model excludes several modifiable risk factors for ROP.

The complexities of ROP pathophysiology are influenced by physical, clinical [8], biochemical [9], genetic and proteomic [10], inflammatory [11,12], and haematological factors [13]. To our knowledge, structural equation modelling has not previously been applied to ROP treatment in order to identify underlying pathophysiological mechanisms for further investigation. The aim of the present study, therefore, is to develop a comprehensive structural model linking physical and clinical risk factors to the need for ROP treatment.

## 2. Material and Methods

### 2.1. Study Population

The sample derived from an anonymised general database, where all the risk factors for premature infants were collected at discharge from 2004 to the present. Children were enrolled following the acquisition of parental written informed consent. Two investigators (GS and TD) extracted data. After obtaining approval from the ethics committee, an anonymised database was obtained in SPSS (OP, GS), where, after applying the selection criteria, we obtained a sample size sufficient for performing structural equation modelling.

The subjects included in this retrospective case–control study were all premature infants born between April 2004 and July 2024 who were screened for ROP. Of the 802 premature infants initially screened, 314 (39.15%) underwent three or more exams with binocular indirect biomicroscopy (BIO). A total of 488 (60.85%) were excluded from this study, as they received only one or two fundus examinations, and hence the data were incomplete. Those who presented with media opacity; had aggressive ROP, stage 4 or 5; and had been treated with anti-VEGF agents were also excluded because the sample was too small [6].

This study was approved by the Biomedical Research Ethics Committee of Andalusia and is registered as No. 0586-N-21 (approval date: 12 March 2021). Signed informed consent was obtained from the parents or caregivers of all infants included in the study.

### 2.2. Ophthalmologic Examination

The following inclusion criteria were applied: gestational age (GA) ≤ 31 weeks or birth weight (BW) < 1500 g or an unstable clinical course as determined by the neonatologist. Premature infants were considered for examination if they meet one or more of the following three criteria: BW between 1501 and 2000 g, GA ≥ 32 weeks, or oxygen supply > 3 days [14]. All infants were examined at postnatal week 4 by a trained paediatric ophthalmologist using BIO with indentation. The follow-up procedures were performed as indicated in the International Classification of Retinopathy of Prematurity [15].

The avascular area of the retina was measured using optic disc diameters (DD), quantified, and recorded in increments of 0.5 DD. Vascularisation rate is defined as the ratio of the avascular area, in the 4th postnatal week, to the time required to vascularise the PAR. The rate of retinal vascularisation (DD/week) was measured from the edge of the avascular area to the retina’s periphery or until the administration of laser treatment [6]. Both measurements were classified as physical risk variables. To obtain this value, at least three fundus examinations were performed.

### 2.3. Categorisation of the Risk Factors for ROP

The vascularisation of the retina advances like a wave from the optic disc to the nasal and temporal periphery. To determine its topographic location, we use the Zones. Zone I consists of a circle centred on the optic nerve with a radius twice the distance from nerve to fovea, or subtending 30 degrees and a radius of 6 papillary diameters [16]. Zone II indicates when the vascularisation has progressed far enough that it has advanced to the nasal retina; it consists of the surface within the circle, with a radius of 12 papillary diameters, and is outside of Zone I. Zone III indicates the crescent temporal retina, the last to vascularise, and consists of a crescent moon measuring 3 papillary diameters.

The plus form is characterised by abnormal tortuosity and dilatation of the retinal blood vessels. The plus form indicates the possibility of rapid worsening. Stages 1 (demarcation line) and 2 (ridge) of acute disease are usually resolved spontaneously. Stage 3 consists of extraretinal neovascular proliferation or flat neovascularisation. Stages 4 and 5 are characterised by partial or complete retinal detachment; for these stages, treatment (pars plana vitrectomy) is required. In this series, only cases treated for classic ROP with a diode laser were included.

The untreated ROP group included infants with a vascularised peripheral retina and those with ROP not requiring treatment (ETROP type 2) [1,15]. Whether or not ROP was treated was considered the dependent variable. Data from mothers and newborns were collected from the analysed hospital’s electronic medical records and always transferred to the same clinical form. The following clinical predictive factors were included: birth weight (g), gestational age (weeks), intubation mechanical ventilation (IMV, days), continuous positive airway pressure (CPAP, days), duration of nasal cannula insertion (days), culture-proven sepsis (with clinical and microbiological confirmation), bronchopulmonary dysplasia, apnoea (characterised by unstable mechanical ventilation and clinical course precluding weekly ROP screening), caffeine administration, weight gain (g/day) at the end of the 4th postnatal week, maternal age, blood transfusion, intraventricular haemorrhage grade (IVH), and necrotising enterocolitis [6]. Measurement of the avascular area and quantification of the vascularisation rate of the retina were performed as described in a previous article [6].

### 2.4. Statistical Analysis

The study groups were compared using univariate analysis. When the data were normally distributed, Student’s *t*-test and ANOVA were applied; the Mann–Whitney U test was used for continuous non-parametric data, and the 2-sided χ^2^ test was used for categorical variables. Univariate analysis was conducted between the dependent variable, i.e., ROP treatment, and the remaining clinical and physical variables. A two-tailed *p* value < 0.05 was considered statistically significant. Odds ratios (ORs) and adjusted ORs (aORs) with 95% confidence intervals (CIs) were calculated. Binary logistic regression analysis was used to assess the association between ROP treatment and significant physical and clinical risk factors identified in the univariate analysis.

Variables were identified through a bivariate analysis. Decision trees and an empirical SEM were developed using SPSS (v29.0, Inc., Chicago, IL, USA; AMOS v26.0, IBM). A large sample size of >200 is appropriate for SEM. Our sample, with 314 premature infants, was thus a large enough sample. In the SEM, ROP was treated as the dependent variable. All analyses were conducted at a 5% significance level. Bivariate associations were included in the SEM only if they were statistically significant and had a correlation greater than 0.3 [17]. A rule-of-thumb criteria overview was adopted to determine the following indices: chi-square (χ^2^, *p* > 0.05), chi-square-to-degrees-of-freedom ratio (2 < χ^2^/df ≤ 3), comparative fit index (CFI > 0.90), root mean square error of approximation (RMSEA, <0.08), standardised root mean square residual (SRMR < 0.08), goodness-of-fit index (GFI, >0.90), and Tucker–Lewis index (TLI > 0.90). These criteria indicated the closeness of the fit to the data [18,19].

## 3. Results

### 3.1. Participant Characteristics

In total, 314 premature infants were included in this study, with an ROP incidence of 43% (*n* = 135). A total of 57 (18.2%) had type 1 ROP (treated), and they were compared to 257 premature infants (81.8%) in the untreated group. Of the former group, all 57 received bilateral laser therapy: 39 had stage 3 Zone II with plus disease, 13 had stage 3 Zone I with plus disease, 4 had stage 2 Zone II with plus disease, and 1 had stage 2 Zone I with plus disease. The median gestational age was significantly lower in the treated ROP group than in the untreated group (27.2 ± 2.1 weeks vs. 29 ± 1.9 weeks) (*p* < 0.001). The median birth weight was also significantly lower in the treated ROP group than in the untreated group (881 ± 256 g vs. 1110 ± 248 g) (*p* < 0.001).

### 3.2. Univariate Analysis of the Association Between Treated ROP and Risk Factors

Table 1 presents the data obtained for physical parameters, clinical risk factors, and comorbidities between neonates with treated ROP and those with untreated ROP, revealing a significant association between ROP treatment and the risk factors.

### 3.3. Decision Tree Analysis of ROP Treatment

**Figure 1:**
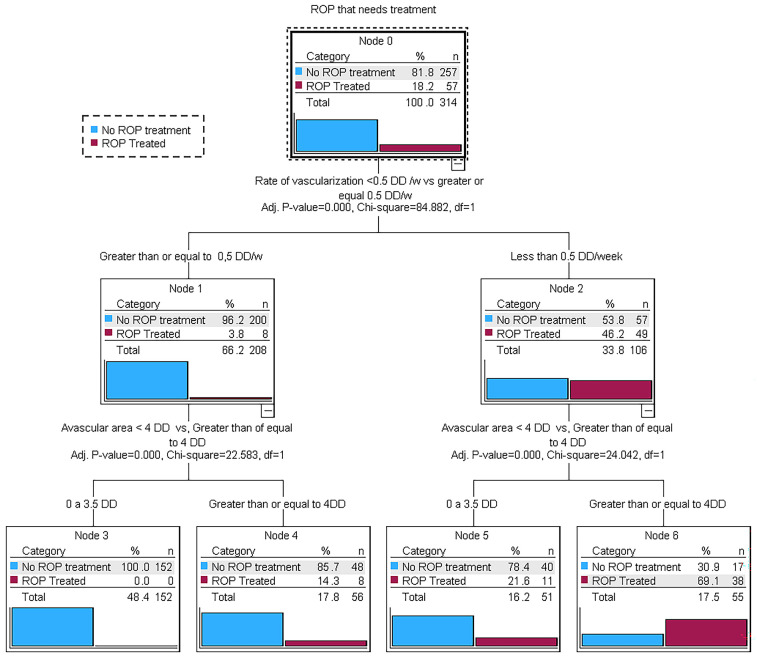
Decision tree analysis of treated ROP.

In the decision tree analysis, a vascularisation rate of <0.5 DD/week was the most significant predictive factor, followed by an avascular area of ≥4 DD as a secondary predictor (Figure 1). The decision tree explained 88.5% of the variance in predicting ROP treatment, with a sensitivity of 66.7% and a specificity of 93.4%.

### 3.4. Multivariate Logistic Regression Analysis of the Association Between Treated ROP and Risk Factors

Multivariate logistic regression analysis identified the following major risk factors for treated ROP: a large avascular temporal area, a weekly vascularisation rate < 0.5 DD/week, and extended IMV for ROP (Nagelkerke’s R^2^ = 64.2%, *p* < 0.000). The model’s sensitivity was 63.2%, with a specificity of 95.2% and an accuracy of 89.5% (Table 2).

The probability of ROP requiring treatment was calculated using the following logistic regression equation:

$$\frac{1}{(1+e^{-(-7.327+0.833(avascular\;area\;in\;DD)+0.051\left(IMV\;time\;in\;days\right)+3.004\;x1(<0.5\;DD/w))}}$$

In this equation, the rate of vascularisation is <0.5 DD/w = 1, ≥0.5 DD/w = 0.

### 3.5. Structural Equation and Risk Factors for ROP Treatment

**Figure 2:**
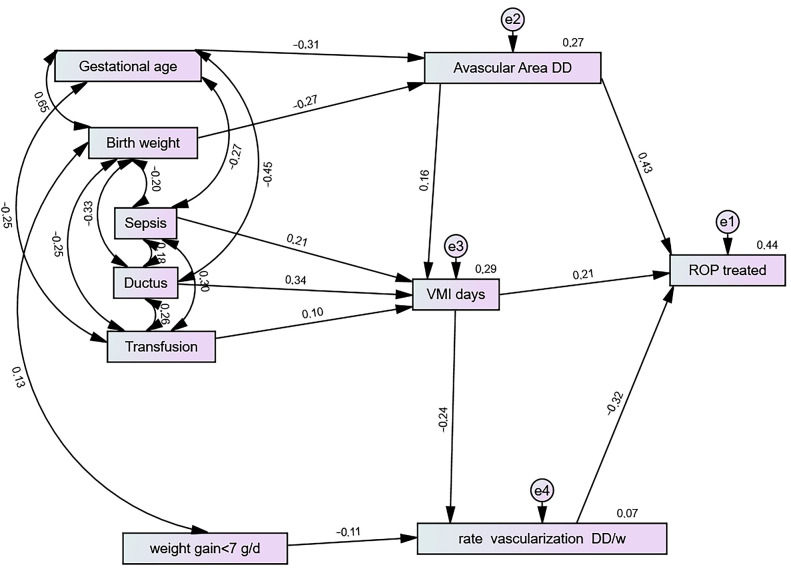
The modelling path diagram above illustrates the final SEM, highlighting the risk factors associated with the need for ROP treatment and revealing interrelationships between the variables. All these relationships were statistically significant (*p* < 0.001), except for those between transfusion and IMV (*p* < 0.049) and between weight gain < 7 g/day and vascularisation rate (*p* < 0.036). Although many variables were significant in the univariate analysis, only those with a correlation above 0.30 were included in the SEM [17]. Several risk factors were included in the final model (left) but excluded from the logistic regression model.

The model demonstrated an adequate-to-good fit for the data: χ^2^ = 46.8, df = 23, *p* = 0.02, χ^2^/gl = 2.03 (≤3–5), CFI = 0.97, RMSEA = 0.058, GFI = 0.97, AGFI = 0.93, and TLI = 0.94 [20,21]. In the final logistic regression model, only the dependent variable (ROP treatment) and three significant associated variables (avascular area in DD, IMV duration, and vascularisation rate) were included. In the SEM (Figure 2), six risk factors on the left side (gestational age, birth weight, sepsis, ductus arteriosus, transfusion, and weight gain < 7 g/day) were also included. These clinical risk variables demonstrated multiple interrelationships that were not accounted for in the logistic regression analysis. However, several risk factors presented correlations or reciprocal relationships, such as gestational age and birth weight, birth weight and weight gain < 7 g/day, and sepsis and transfusion.

The model explained 44% of the variance in ROP treatment, with the remainder accounted for by the latent variable e1. Three intermediate variables had a direct effect on the need for ROP treatment: the vascular area at the fourth postnatal week (which accounted for 43% of this variance), a reduced weekly vascularisation rate (32%), and a shorter duration of IMV (21%) (in all cases, *p* < 0.001). Thus, the six external variables on the left exerted an indirect effect on the need for ROP treatment.

### 3.6. Avascular Area (DD)

A greater avascular area (DD) is positively correlated with IMV and explains 16% of the variance. It is negatively correlated with gestational age, explaining −31% of the variance, and birth weight, explaining −27% of the variance, with *p* < 0.001 in all cases.

Four variables have an indirect effect on greater avascular area (DD). Since gestational age and birth weight are highly correlated, only the variable with the greatest effect was included. The indirect effects of sepsis, ductus arteriosus, and transfusion on the extension of the avascular area across gestational age (Figure 2) are as follows.

Sepsis (−0.27 × −0.31 = 8.4%) variance: greater avascular area in DD vs. non sepsis.

Ductus (−0.45 × −0.31 = 14%): larger DD. 

Transfusion (−0.25 × −0.31 = 7.7%): larger DD.

A case where a premature infant has these three risk factors instead of none can predict a 30% greater temporal avascular area, initiating screening examinations at 4 weeks after birth.

### 3.7. Vascularisation Rate (DD/w)

The vascularisation rate (VR) in DD/w is negatively correlated with the duration of IMV, explaining 24% of the variance (*p* < 0.001), and a postnatal weight gain of <7 g/d, explaining −11% of the variance (*p* < 0.036).

The indirect effects of sepsis, ductus arteriosus, and transfusion on vascularisation rate (VR) (DD/w) across invasive mechanical ventilation (Figure 2) are as follows:

Sepsis (0.21 × −0.24 = −5%) variance of the lower VR vs. non sepsis;

Ductus (0.34 × −0.24 = −8.2%) of the lower VR;

Transfusion (0.10 × −0.24 = −2.4%) of the lower VR.

The presence of these three risk factors (as opposed to their absence) in a premature infant can indicate a 15.6% lower vascularisation rate at the third ROP examination performed between the 6th and 8th postnatal week.

Gestational age correlates positively with birth weight, which explains 65% of the variance; moreover, it is negatively correlated with sepsis, which explains 27% of the variance in its presentation, and ductus arteriosus, which explains 45% of the variance in its presentation. Finally, the need for transfusion explains 25% of the variance in its presentation. All these associations are significant (*p* < 0.001). The younger the gestational age, the greater the risk of ductus arteriosus, sepsis, and the need for transfusion.

Birth weight is negatively correlated with sepsis, explaining −10% of its variance; ductus explains −33% of the variance, and the need for transfusion explains −25% of its variance, with these associations being significant (*p* < 0.001) in every case. The lower the birth weight, the higher the risk of sepsis, ductus, and the need for transfusion.

The presence of sepsis, ductus arteriosus, and the need for transfusion explains 21%, 34%, and 10%, respectively, of the variance in the total duration of IMV. However, these variables are closely associated with each other: thus, sepsis–ductus arteriosus accounts for 18% of the common variance, sepsis–transfusion accounts for 30%, and ductus–transfusion accounts for 26%.

## 4. Discussion

Determining the location of ROP by zone and the posterior extent of retinal avascularity may help identify infants at the highest risk of requiring ROP treatment [22,23]. Low birth weight and gestational age are significant risk factors for the development of avascular retina [24]. During the first postnatal screening, younger gestational age is associated with a larger avascular area in the retina. Additionally, multiple risk factors contribute to further vascular delay and an increased temporal avascular area in the retina [25]. These include the presence of sepsis, the presence of ductus arteriosus, and the need for blood transfusions, and all these variables interact indirectly with gestational age and birth weight.

The vascularisation rate of the retina decreases as the ROP stage advances [26,27], and a rate below 0.5–0.54 DD/week increases the risk of ROP treatment being required [7]. In the SEM, a vascularisation rate below 0.5 DD/week was associated with a prolonged duration of IMV and poor postnatal weight gain (<7 g/day).

If the area of peripheral retina that should be vascularised in a given postnatal week remains ischemic, either because the initial avascular area was greater or because the vascularisation rate is insufficient, the risk of ROP progression increases, as does the need for ROP treatment. Our decision tree analysis for ROP treatment identified two key predictive variables: an avascular area greater than 4 DD and a vascularisation rate below 0.5 DD/week.

Our logistic regression model incorporated an additional variable: duration of IMV. Both models included physical variables (avascular area and vascularisation rate), making them suitable for integration into retinographs that use artificial intelligence for image capture, composition, and predictive risk calculations.

In children with a gestational age (GA) of less than 29 weeks, the rate of treatment-warranted ROP increases with each episode of sepsis [5]. In the SEM adjusted for GA, sepsis, ductus arteriosus, and the need for transfusion were associated with an increased duration of IMV. A clear and statistically significant relationship between oxygen exposure and ROP severity was recorded [4]. Prolonged IMV is considered the strongest predictor of ROP progression and the need for treatment [28]. Thus, for each additional day of oxygen administration, the risk of ROP progression increases by 1% [29].

The SEM also integrated six clinical risk factors: GA, BW, sepsis, ductus arteriosus, transfusion, and weight gain < 7 g/day. These factors interact, generating both direct and indirect effects on the model’s three intermediate variables: extension of the avascular area at the fourth postnatal week, total IMV duration, and vascularisation rate from the fourth to eighth postnatal week. The SEM enabled us to integrate modifiable risk factors into the ROP treatment model, revealing that the assumed independence of these risk factors does not hold, as the variables are highly interrelated.

Clinical investigation also revealed a complex network of interconnected factors involved in the pathogenesis of ROP [30]. Thus, both cytokines and growth factors were present in the analysis networks. Among infants with ROP treatment, the presence of diseases such as sepsis, ductus, anaemia, or bronchopulmonary dysplasia provokes changes in ROP-related networks [31], the extension of the avascular area, and the rate of vascularisation.

This study is subject to certain limitations. Firstly, at least three examinations were required to determine the vascularisation rate of the retina, which probably limited the sample to more severe ROP cases. Furthermore, perinatal and ophthalmologic care have experienced significant change over the last 20 years, a factor that is not considered in our analysis. Finally, this study was conducted at a single tertiary health centre; in future research, the findings reported should be validated with data from other sources. A key strength of this study, however, is its integration of multiple known risk factors into a general model using structural equation modelling.

With respect to the need for ROP treatment, the structural equation model explains less variance than logistic regression (44% vs. 64%); the rest can be explained by the indirect effects of the variables of the model, the latent variable e1, and heritability. If we start from a heritability of retinopathy of prematurity of 70–72%, the heritability of the risk of ROP treatment would be around 18% (unpublished data). The structural equation shows that there is a progressive drift from hereditary factors to environmental factors or clinical decisions at the time of ROP treatment, allowing a wide margin for improvement in ROP care.

However, the SEM suggests that the supposed independence of the risk factors is not real, and that, in fact, these variables are strongly interrelated. The SEM also enables us to include numerous modifiable risk factors in a more general predictive model, thus facilitating the inclusion of new risk factors and cytokines as future research directions. As possible applications of the research we describe, multivariate causal relationships could be evaluated. Another useful area for study is the relationships among certain physical variables related to vascularisation, namely, the extension of the avascular area, the rate of vascularisation, and their direct and indirect relationships with modifiable or non-modifiable clinical risk factors.

To reduce the need for treatment for ROP, ophthalmologists and paediatricians must address modifiable risk factors. The structural equation model we present enables the joint consideration of physical and clinical variables and reveals associations between risk factors that might not be detectable through decision trees and logistic regression alone. Structural equation modelling allows us to determine how numerous risk variables interact dynamically and simultaneously via multiple pathways.

Lower gestational age and birth weight are associated with several exogenous risk factors, which explain their importance in predictive models of ROP treatment [32]. In our SEM model, GA and BW exerted an indirect effect on the need for ROP treatment through an increase in ischemic avascular area.

## 5. Conclusions

Lower gestational age, lower birth weight, sepsis, ductus arteriosus, transfusion, and lower weight gain increase the risk of requiring ROP treatment. In the SEM, this phenomenon is represented through three intermediate physical endogenous variables, namely, a greater temporal avascular area of the retina, a lower postnatal vascularisation rate, and a greater duration of IMV. However, the validity of this predictive model needs to be confirmed in further research, conducted at other health centres.

## Figures and Tables

**Table 1:** Univariate analysis of risk factors for developing ROP requiring treatment. Disk diameter (DD) and week (w).

Risk Factor	*p* Value	Odds Ratio (95% CI)	R^2^ Nagelkerke
Each additional diameter of avascular area (DD)	0.000	2.32 (1.89–2.9)	38.6%
Retinal vascularisation rate, <0.5 vs. ≥0.5 DD/w	0.000	21.05 (9.6–48)	38.1%
Duration of invasive respiratory support (days)	0.000	1.075(1.05–1.1)	23.0%
Birth weight (g)/100	0.000	0.65 (0.56–0.76)	19.7%
Apnoea	0.000	9.7 (4.7–19.7)	19.1%
Gestational age (weeks)	0.000	0.62 (0.53–0.73)	18.3%
Number of associated comorbidities	0.000	1.23 (1.12–1.34)	10.9%
Hydrocephalus shunt	0.000	13.8 (3.5–54)	8.3%
Indomethacin	0.000	4.3 (2.1–8.6)	8.0%
Patent ductus arteriosus	0.000	3.3 (1.17–10.1)	7.4%
Hyaline membrane ≥ 3	0.000	3.1 (1.7–5.7)	7.1%
Brain haemorrhage (yes/no)	0.000	3.6 (1.8–7.2)	6.6%
Transfusion	0.001	2.2 (1.32–3.07)	6.0%
Sepsis	0.001	2.6 (1.4–4.7)	5.4%
High-flow nasal cannula (days)	0.002	1.03 (1.01–1.04)	4.9%
Weight gain, <7 g/day, 4th postnatal week	0.005	2.7 (1.3–5.5)	3.7%
Surfactant treatment	0.024	1.96 (1.09–3.5)	2.6%

**Table 2:** Logistic regression analysis of risk factors for treated ROP.

Risk Factors	*p* Value	Odds Ratio (95% CI)
Duration of invasive respiratory support, days	0.001	1.05 (1.02–1.08)
Each additional diameter of avascular area (DD)	0.000	2.3 (1.7–3.0)
Retinal vascularization rate, <0.5 vs. ≥0.5 DD/week	0.000	20.2 (7.3–55.6)

Abbreviations: ROP: retinopathy of prematurity; DD: diameter of the disc.

## Data Availability

The authors declare that the data in this research are available from the corresponding authors upon reasonable request.
